# Early neurodevelopmental problems and risk for avoidant/restrictive food intake disorder (ARFID) in 4‐7‐year‐old children: A Japanese birth cohort study

**DOI:** 10.1002/jcv2.12094

**Published:** 2022-08-07

**Authors:** Lisa Dinkler, Kahoko Yasumitsu‐Lovell, Masamitsu Eitoku, Mikiya Fujieda, Narufumi Suganuma, Yuhei Hatakenaka, Nouchine Hadjikhani, Rachel Bryant‐Waugh, Maria Råstam, Christopher Gillberg

**Affiliations:** ^1^ Gillberg Neuropsychiatry Centre Institute of Neuroscience and Physiology University of Gothenburg Gothenburg Sweden; ^2^ Department of Environmental Medicine Kochi Medical School Kochi University Nankoku, Kochi Japan; ^3^ Department of Pediatrics Kochi Medical School Kochi University Nankoku, Kochi Japan; ^4^ Faculty of Humanities and Social Sciences University of the Ryukyus Nishihara, Okinawa Japan; ^5^ Athinoula A. Martinos Center for Biomedical Imaging Massachusetts General Hospital Harvard Medical School Charlestown Massachusetts USA; ^6^ Maudsley Centre for Child and Adolescent Eating Disorders South London and Maudsley NHS Foundation Trust London UK; ^7^ Department of Child and Adolescent Psychiatry Institute of Psychiatry Psychology and Neuroscience King's College London London UK; ^8^ Department of Clinical Sciences Lund Lund University Lund Sweden; ^9^ Department of Psychiatry Kochi Medical School Kochi University Nankoku, Kochi Japan

**Keywords:** attention‐deficit/hyperactivity disorder, autism spectrum disorder, eating disorder, Japan Environment and Children's Study (JECS), neurodevelopmental disorders

## Abstract

**Background:**

An overrepresentation of neurodevelopmental problems (NDPs) has been observed in individuals with avoidant/restrictive food intake disorder (ARFID). Previous studies on the association between ARFID and NDPs have been limited by cross‐sectional data from clinical samples of small size. This study aimed to extend previous research by using prospectively collected data in a non‐clinical child cohort. We examined the occurrence of early NDPs in 4–7‐year‐old children with suspected ARFID and how predictive early NDPs are of ARFID.

**Methods:**

Data were collected via parent‐report a sub‐sample of the Japan Environment and Children's Study (JECS) including 3728 children born 2011–2014 in Kochi prefecture. NDPs were assessed biannually between 0.5 and 3 years of age with the Ages and Stages Questionnaire‐3, at age 2.5 years with the ESSENCE‐Q, and at age 1 and 3 years via parent‐reported clinical diagnoses. ARFID was identified cross‐sectionally (at age 4–7 years) using a newly developed screening tool. Logistic regressions were used to test association of (1) a composite early NDP risk score, (2) specific early NDPs, and (3) neurodevelopmental trajectories over time with ARFID.

**Results:**

Children in the highest risk percentiles of the NDP risk score had roughly three times higher odds of having suspected ARFID; the absolute risk of later ARFID for children above the 90th percentile was 3.1%. Early NDPs (excluding early feeding problems) were more predictive of later ARFID than were early feeding problems. Specific NDPs predictive of ARFID were problems with general development, communication/language, attention/concentration, social interaction, and sleep. Neurodevelopmental trajectories of children with and without suspected ARFID started to divert after age 1 year.

**Conclusions:**

The results mirror the previously observed overrepresentation of NDPs in ARFID populations. In this non‐clinical child cohort, early feeding problems were common and rarely developed into ARFID; however, our findings imply that they should be monitored closely in children with high NDP risk to prevent ARFID.

## Graphical abstract

In our sample of 3728 Japanese children from a non‐clinical cohort, circa 3% of children with neurodevelopmental problems between age 0.5 and 3 years screened positive for avoidant/restrictive food intake disorder (ARFID) between age 4 and 7 years. This reflects a three times increased risk of ARFID in children with early neurodevelopmental problems and suggests that early neurodevelopmental problems might aid in the early detection of ARFID. Neurodevelopmental trajectories of children with and without suspected ARFID started to divert after age 1 year.

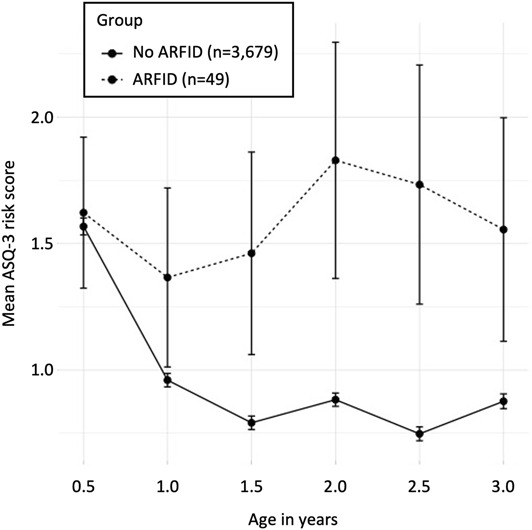

**Key points**

- Cross‐sectional studies have shown that neurodevelopmental disorders are more common in individuals with avoidant/restrictive food intake disorder (ARFID).
- We examined how predictive early neurodevelopmental problems are of later ARFID using prospectively collected data in a non‐clinical child cohort.
- Circa 3% of the children with significant early neurodevelopmental problems screened positive for ARFID between age 4 and 7 years, reflecting a three times increased risk of ARFID compared to children without significant early neurodevelopmental problems.
- Early feeding problems were common but rarely developed into ARFID; early neurodevelopmental problems were better predictors of later ARFID.
- Early neurodevelopmental problems might aid in the early detection of ARFID. Children with feeding and neurodevelopmental problems should be monitored closely for the development of ARFID.

## INTRODUCTION

Avoidant/restrictive food intake disorder (ARFID) is characterized by a persistent restriction of food intake in amount and/or variety that results in weight loss or failure to gain weight, insufficient growth, nutritional deficiency, dependence on enteral feeding or oral nutritional supplementation, and/or marked interference with psychosocial functioning (American Psychiatric Association, 2013). Contrary to other eating disorders such as anorexia nervosa and bulimia nervosa, ARFID is not motivated by body image concerns or drive for thinness. Instead, food avoidance/restriction in ARFID is often based on one or more of three “drivers”: (1) concern about aversive consequence of eating (e.g., choking, vomiting), (2) sensory‐based avoidance (e.g., based on the smell, taste, appearance, or consistency/texture of foods), and (3) lack of interest in food or eating (American Psychiatric Association, 2013).

A higher than expected occurrence of neurodevelopmental problems (NDPs) and neurodevelopmental disorders (NDDs) has been observed in patients with ARFID compared to general population estimates, with some studies identifying higher rates of co‐occurrence with ARFID than with anorexia nervosa (Lieberman et al., 2019; Nicely et al., 2014; Norris et al., 2021). In children and adolescents with ARFID treated at feeding/eating disorder clinics, the prevalence of specific NDDs has been estimated at 3%–23% for autism spectrum disorder (ASD) (Kambanis et al., 2020; Lieberman et al., 2019; Norris et al., 2021; Reilly et al., 2019), 3%–39% for attention‐deficit/hyperactivity disorder (ADHD) (Duncombe Lowe et al., 2019; Lieberman et al., 2019; Nicely et al., 2014; Norris et al., 2021; Reilly et al., 2019), 10%–31% for learning difficulties/disorders (Lieberman et al., 2019; Nicely et al., 2014; Norris et al., 2021), and 26%–38% for intellectual disability or general developmental delay (Nicely et al., 2014; Sharp et al., 2020). Mechanisms underlying the observed ARFID‐ASD comorbidity might, for instance, comprise common ASD symptoms such as sensory sensitivity, repetitive behaviours, rigidity, and high arousal, which individually or together might predispose the child to developing ARFID. In children with ADHD, difficulties remaining seated at meals and keeping focused on eating due to attention difficulties and high activity levels might be predisposing factors for ARFID.

To the best of our knowledge, research on ARFID and NDPs has so far almost exclusively been of a cross‐sectional nature and limited to specific clinical samples from the US and Canada with small sample sizes. Clinical samples might be biased in that children with more severe ARFID, potentially caused by multi‐comorbidity, might be overrepresented. The only larger study that we have been able to locate estimated that in a cohort of 5157 individuals with ASD—largely identified through clinical sites—21% were at high risk for ARFID (Koomar et al., 2021). Furthermore, the recognition of the ARFID diagnosis is relatively recent and referral routes have often not yet been standardized. Children with ARFID might therefore be encountered in a range of different specialties (e.g., general practice, paediatrics, psychiatry, gastroenterology, dietetics, occupational therapy). For instance, while children with ARFID and comorbid medical conditions might be referred to paediatric clinics, those with ARFID and considerable fear/anxiety may be more likely to be referred to child and adolescent mental health services (CAMHS). The estimated prevalence of NDP/NDD comorbidity might therefore depend heavily on the specific speciality that a sample has been drawn from. In contrast, samples screened from the general population are potentially more representative of the entire group of individuals affected by ARFID, including those who are not seeking treatment. To date, there have been no studies examining the possible association of ARFID and NDPs in non‐clinical samples.

It has been well‐established that early NDPs are highly predictive of later diagnosed NDDs and that the early recognition of such problems aids in the early detection of children with NDDs, which, in turn, will enable early interventions (Gillberg, 2010; Hatakenaka et al., 2016; Stevanovic et al., 2018). These early symptoms include, for instance, motor abnormalities, speech and language delay, abnormal sensory reactions, inattention, overactivity, and sleep problems (Gillberg, 2010). Early feeding problems are also included in this range of NDPs potentially indicating the presence of NDDs (Gillberg, 2010), and in children with ASD, feeding problems often constitute one of the first problems parents worry about and seek help for (Barnevik Olsson et al., 2013). Due to the suggested significant overlap between ARFID and NDDs, and the fact that children with NDDs are not always diagnosed or often diagnosed very late (Gould & Ashton‐Smith, 2011; Huang et al., 2020), the question arises whether early NDPs can also aid in the early detection of ARFID. This has not previously been examined using longitudinal data.

### Aim of this study

The present study extends previous research on ARFID and NDPs/NDDs by using prospectively collected data in children from a non‐clinical sample. We aimed to examine the occurrence of early NDPs in 4–7‐year‐old children with suspected ARFID and how predictive early NDPs are of ARFID. First, we examined risk of ARFID in children with early NDPs and to what extent the presence of early NDPs predicts later ARFID. Hereby, we excluded early feeding problems from the NDP measure used. We hypothesized that children with early NDPs would be at *increased risk* for ARFID. Second, we tested the association of *specific* early NDPs with ARFID. Based on previous findings, we expected that specifically early NDPs related to ASD, ADHD, intellectual disability, and learning difficulties would be associated with later ARFID. Given the central role of sensory‐based avoidance in individuals with ARFID (Reilly et al., 2019), we also hypothesized that early sensory sensitivity would be predictive of ARFID. Finally, we hypothesized that most children with suspected ARFID would show feeding problems early on. In addition, as an exploratory aim, we examined at what age children with suspected ARFID on average start to divert in their neurodevelopment from children without ARFID.

## METHODS

### Study population

We conducted a cross‐sectional parental survey in a sub‐sample of the Japan Environment and Children's Study (JECS), using a screening tool for ARFID recently developed by our group (Dinkler et al., 2022). JECS is an ongoing nationwide birth cohort study, investigating environmental factors affecting children's health and development (Kawamoto et al., 2014; Michikawa et al., 2018). Our study was conducted in collaboration with the Kochi Regional Centre of JECS at Kochi Medical School. In December 2018, the survey was sent out to the parents of 6633 JECS participants in Kochi prefecture, born between July 2011 and December 2014. The response rate was 56.5% (*n* = 3746). After excluding 18 children due to missing/unclear responses relating to ARFID criteria, the final sample consisted of 3728 children. Attrition analyses showed that, compared to non‐responding mothers, responding mothers were more highly educated, had a higher annual household income, and smoked less often during pregnancy, but few differences were found regarding maternal health variables except that responding mothers were less often overweight prior to pregnancy and experienced less psychological distress during pregnancy. Children of responders and non‐responders did not differ significantly in gestational age, birth weight, or Apgar score (see Supplement 1 in (Dinkler et al., 2022)). The study was approved by the Ethics Committee at Kochi Medical School (ERB‐102925 and ERB‐104083). Informed consent was obtained from all participants.

### Measurements

#### ARFID screening

Avoidant/restrictive food intake disorder was assessed using a newly developed, parent‐reported screener for children aged 2 years and up. The development and contents of the ARFID screener are described in Dinkler et al. (2022). In short, the screener contains 10 items that closely map onto the DSM‐5 diagnostic criteria for ARFID. Items, response options, and screening algorithm are shown in Table S1. In addition, the three drivers of food avoidance/restriction in ARFID were measured with one item each (Table S1). For details regarding the translation process please refer to Dinkler et al. (2022). The ARFID screener has shown satisfactory convergent validity with problems related to mealtime behaviours, nutritional intake, selective eating, and satiety responsiveness, as well as with shorter height and lower body mass index (Dinkler et al., 2022). Children were identified as screening positive for ARFID when they met DSM‐5 ARFID criterion A, plus at least one of criteria A1, A2, A3, and A4, as well as criteria C and D. Criterion B (the eating disturbance is not due to lack of available food or a culturally sanctioned practice) was not assessed as the majority of our cohort was considered (a) affluent enough for food shortage to be relatively unlikely, and (b) culturally homogenous enough with no particular food restriction practice. For reasons of readability, children screening positive for ARFID are referred to as *children with suspected ARFID,* and children screening negative for ARFID are referred to as *children without suspected ARFID* in the following.

#### Prospective assessment of NDPs (before ARFID screening)

**FIGURE 1:**
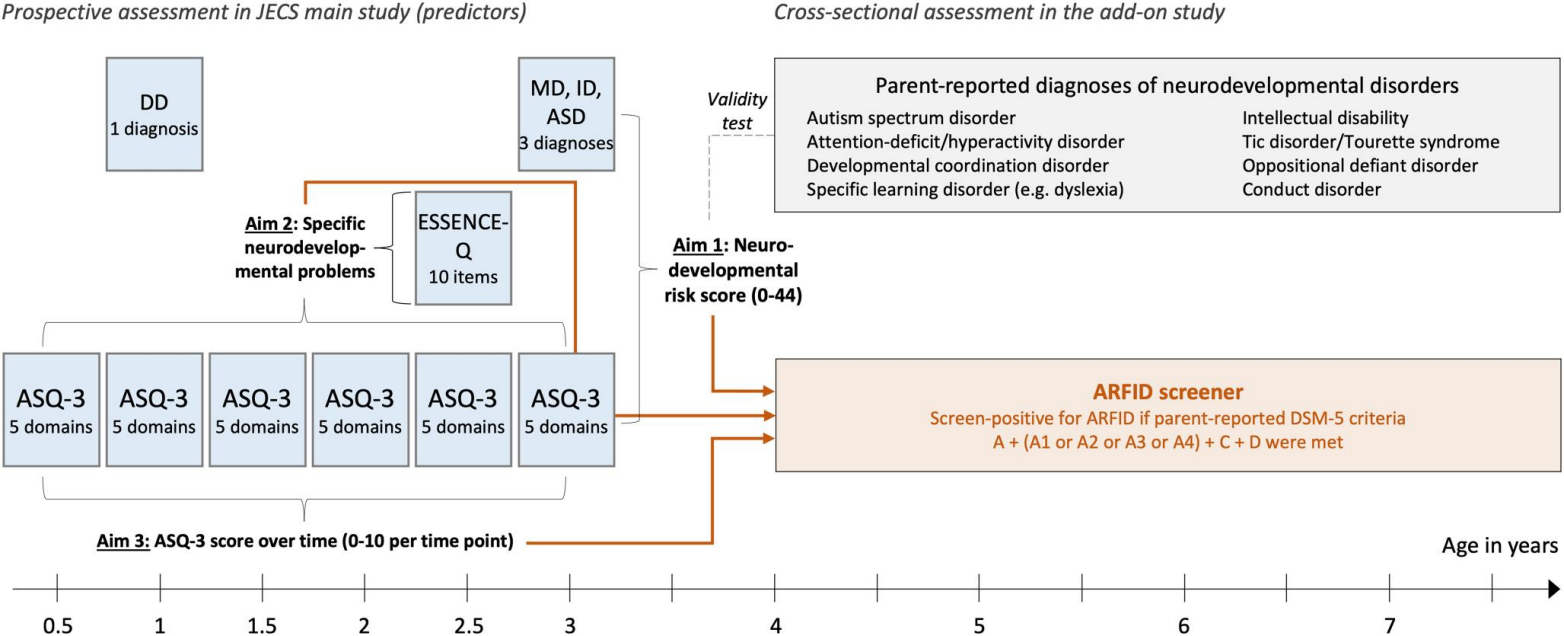
Overview of assessments, predictors, and outcomes in the current study. Predictors were derived as follows. 1. Neurodevelopmental risk score. Score below the referral cut‐off on an Ages and Stages Questionnaire‐3 (ASQ‐3) domain: 1 risk point (6 time points of measurement on 5 ASQ‐3 domains: max. 30 points) + ESSENCE‐Q items (except feeding problems), rated with “yes”: 1 risk point (10 items: max. 10 risk points) + parent‐reported diagnoses of developmental delay (DD; at age 1 year), motor delay (MD), intellectual disability (ID) or autism spectrum disorder (ASD; at age 3 years: 1 risk point per diagnosis (4 diagnoses: max. 4 risk points) = theoretical range 0–44. 2. Specific Neurodevelopmental problems. Each of 10 ESSENCE‐Q items and each ASQ‐3 domain that was failed 2 or more out of 6 time points of measurement. 3. ASQ‐3 score. Score below monitoring cut‐off: 1 risk point/domain, score below referral cut‐off: 2 risk points/domain, × 5 domains = 0–10 per time point

Data on NDPs came from different sources (Figure 1). As part of the JECS main study, the Japanese version of the **Ages and Stages Questionnaire‐3** (Mezawa et al., 2019; J‐ASQ‐3; Squires & Bricker, 2009) was collected every 6 months after birth. Data were available until the age of 3 years for the current study. The ASQ‐3 assesses parent‐reported developmental delay in five skill domains: communication (language skills), gross motor (e.g., sitting, crawling, walking, running), fine motor (hand and finger movement/coordination), problem‐solving (e.g., playing with toys) and personal‐social (self‐help skills and interaction). Each domain consists of six questions on whether a certain activity can be done by the child, rated with “yes” (10 points), “sometimes” (5 points), or “not yet” (0 points). Two cut‐off values exist for the resulting domain scores at each age to identify children potentially at risk for developmental delay: a “monitoring cut‐off” at 1 standard deviation (SD) below the mean and a “referral cut‐off” at 2 SD below the mean (Mezawa et al., 2019).

We also used data from the Japanese version of the **ESSENCE‐Q** (Early Symptomatic Syndromes Eliciting Neurodevelopmental Clinical Examinations Questionnaire; Hatakenaka et al., 2016; see also Cederlund, 2022; Stevanovic et al., 2018), which was collected in the JECS main study at child age 2.5 years. The ESSENCE‐Q screens for a broad range of early NDPs that might indicate the presence of NDDs and therefore suggest the need for clinical examination. The ESSENCE‐Q is intended for use in both clinical practice and epidemiological research and consists of 11 short questions all starting with “Have you (or anybody else, who?) been concerned for more than a few months regarding the child's […]”. The exact wording of the 11 NDP areas can be found in Table 2. It can be used as a questionnaire or as a short interview conducted by a clinician. Questions are rated with “yes”, “maybe/a little” or “no”. The ESSENCE‐Q has high sensitivity and relatively low specificity, which is appropriate for a screening instrument. It has been tested as a parent questionnaire in routine Japanese public child health settings (<age 4), where area under the curve (AUC) values between 0.63 and 0.80 (Hatakenaka et al., 2017; Hatakenaka et al., 2020). In the JECS, the response format was changed to “yes” or “no”. The validity of this adjustment has not been investigated.

In addition, parents were asked at child age 1 year whether their child had been diagnosed by a doctor with developmental delay, and at child age 3 years whether their child had been diagnosed by a doctor with motor delay, intellectual disability, or ASD. Parents were also requested to indicate the name of the clinic where the child was diagnosed, so that doctors could be contacted to confirm the diagnoses.

We derived a **NDP risk score** by aggregating the above described measures as follows: scoring below the referral cut‐off on an ASQ‐3 domain—1 risk point (6 time points of measurement on 5 ASQ‐3 domains: max. 30 risk points); ESSENCE‐Q items except feeding problems, rated with “yes”—1 risk point (10 items: max. 10 risk points); parent‐reported diagnoses of developmental delay (at age 1 year), motor delay, intellectual disability or ASD (at age 3 years)—1 risk point per diagnosis (4 diagnoses: max. 4 risk points). In total, the NDP risk score had a theoretical range from 0 to 44. Feeding problems were not included into the NDP risk score so we could examine to which degree cumulative NDP risk predicted later ARFID over and above previous feeding problems.

To make use of the repeated measurements of the ASQ‐3 between 0.5 and 3 years of age, we computed individual **ASQ‐3 risk scores** for each time point of measurement as follows: scoring below the monitoring cut‐off on an ASQ‐3 domain—1 risk point; scoring below the referral cut‐off on an ASQ‐3 domain—2 risk points. Summed up over the five ASQ‐3 domains, this yielded an individual ASQ‐3 risk score with a theoretical range of 0–10 points per time point of measurement.

#### Cross‐sectional assessment of NDD diagnoses (concurrent with ARFID screening)

In the survey that was sent out to parents at child age 4–7 years, parents were also asked to indicate whether their child had received a NDD diagnosis, including ASD, ADHD, developmental coordination disorder, intellectual disability, tic disorder/Tourette syndrome, specific learning disorder (e.g. dyslexia), oppositional defiant disorder, and conduct disorder.

#### Validity of the NDP risk score

Apart from analysing the continuous NDP risk score, we also created binary variables comparing those with highest NDP risk (i.e., scoring *above* the 80th, 90th, 95th and 99th percentile) to those with lower NDP risk (i.e., scoring *below* the 80th, 90th, 95th and 99th percentile). The prevalence of NDDs in the general child population is roughly 10% (Gillberg, 2010). Children scoring above the 90th percentile on the NDP risk score are, therefore, likely to approximately represent the population with NDDs. Similarly, those scoring above the 80th percentile are likely at increased risk of NDDs, while those above the 95th and 99th percentile will almost certainly have one or several NDDs. To investigate the validity of the NDP risk score we calculated the odds of having any diagnosed NDD measured cross‐sectionally at age 4–7 years for children scoring above the 80th, 90th, 95th and 99th percentiles of the NDP risk score. Odds ratios were as follows: above 80th percentile: OR = 10.08 (95% CI 7.05–14.39); above 90th percentile: OR = 13.71 (95% CI 9.62–19.53); above 95th percentile: OR = 16.62 (95% CI 11.36–24.31); above 99th percentile: OR = 31.17 (95% CI 16.68–58.25). Although confidence intervals were large, these data show that the NDP risk score used in this study is a good approximation of risk for NDDs.

#### Statistical analyses

Group differences in sample characteristics were tested with chi square tests for categorical outcomes and Welch's *t*‐tests for continuous outcomes. We used logistic regressions with ARFID status (screen‐positive vs. screen‐negative for ARFID) as the dependent variable for all three aims to test association of ARFID status with NDP risk score (Aim 1), specific NDPs (Aim 2), and ASQ‐3 risk score at different ages (Aim 3). To corrected for multiple testing in Aims 2 and 3, we calculated *Q* values using the false discovery rate (FDR) approach at an FDR of 0.05 (Benjamini & Hochberg, 1995; Benjamini & Yekutieli, 2001). Stata 16.1 was used for data analysis (StataCorp, 2019) and R 4.0.0 for data visualization (R Core Team, 2020).

## RESULTS

Demographic and birth‐related characteristics are presented in Table 1. No significant differences emerged between those with and without ARFID; these variables were therefore not included as covariates in the main analyses. Almost all questionnaires were completed by mothers (98.1%). Forty‐nine children (1.3%) were identified with ARFID (22 boys, 27 girls; see Dinkler et al., 2022). The frequency of specific diagnostic criteria in children with ARFID can be found in Table S1. Lack of interest in food or eating (63.3%) and sensory‐based avoidance (51.0%) were the most common drivers of food avoidance, while concern about aversive consequences of eating was less common (14.3%).

**TABLE 1:** Demographic and birth‐related characteristics in the sample (*n* = 3728)

	Total sample (*n* = 3728)	ARFID (*n* = 49)	No ARFID (*n* = 3679)
Characteristic	%		
Sex, % female	49.1	55.1	49.0
Age in months cross‐sectional follow‐up, mean (SD), median (range)	68.1 (11.0), 67 (49–95)	67.7 (12.3), 66 (50–88)	68.1 (11.0), 67 (49–95)
Gestational age at birth in weeks, %			
Total, mean (SD)	39.1 (1.7)	39.3 (1.5)	39.1 (1.6)
Preterm births (<37)	5.3	4.1	5.3
Term births (37–41)	94.3	95.9	94.3
Post term births (≥42)	0.4	0.0	0.4
Birth weight in g, mean (SD)	2987 (414)	2928 (457)	2987 (413)
Multiple births, %	1.3	2.0	1.3
Apgar score <7 at 1 min after birth, %	2.5	2.1	2.5
Apgar score <7 at 5 min after birth, %	0.8	0.0	0.8
Maternal age at study entry (during trimester 1), %			
Total, mean (SD)	31.2 (4.7)	31.7 (5.0)	31.2 (4.7)
<25	7.3	6.1	7.3
25–29	29.9	28.6	30.0
30–34	37.1	34.7	37.0
≥35	25.7	30.6	25.7
Maternal education in years, %			
<10	3.1	6.4	3.1
10–12	23.3	23.4	23.3
13–16	71.9	68.1	71.9
≥17	1.7	2.1	1.7
Annual household income in million Japanese Yen, %			
<2	7.5	6.5	7.5
2 to <4	33.6	41.3	33.5
4 to <6	33.0	28.3	33.0
6 to <8	18.0	19.6	18.0
8 to <10	5.2	4.4	5.2
≥10	2.8	0.0	2.9
Any person smoking in the household, %	19.9	30.6	19.7

*Note*: Differences between groups were tested with chi square tests for categorical outcomes (*sex, preterm/term/post term birth, multiple birth, Apgar score* < *7 at 1 and 5 min, maternal age in groups, maternal education, annual household income, smoking in the household*), and Welch's *t*‐tests for continuous outcomes (*age, gestational age, birth weight, maternal age*). All *p*‐values were >0.3, except for smoking in the household (*p* = 0.06).

### (1) To what extent do early NDPs predict ARFID?

Logistic regression showed that the higher the NDP risk score (including repeated measures between 0.5 and 3 years of age but excluding early feeding problems) the higher the risk for ARFID measured cross‐sectionally when children were between 4 and 7 years of age. The odds for ARFID increased by 11% for each unit increase on the NDP risk score (theoretical range: 0–44; Table 2). Children in the highest risk percentiles had roughly three times higher odds of having ARFID, except for those above the 99th percentile, where the odds for having ARFID were 8.43, however with a broad confidence interval. The absolute risks of later ARFID for children above the 80th (90th, 95th, 99th) percentile on the NDP risk score were 2.5% (3.1%, 3.7%, 9.3%) compared to an ARFID prevalence of 1.3% in the total sample. A fifth (20.8%) of children with ARFID had a NDP risk score above the 90th percentile, indicating the presence of one or more NDDs, compared to 8.6% of children without ARFID (OR = 2.80, 95% CI 1.38–5.67: Table 2). The NDP risk score predicted later ARFID better (pseudo *R* ^2^ = 3.2%, *p* = 0.0001) than when early feeding problems were used as the only predictor of ARFID (pseudo *R* ^2^ = 1.8%, *p* = 0.003). Both predictors together explained 4.4% of the variance of later ARFID.

**TABLE 2:** Longitudinal/prediction: Early NDPs at age 0.5‐3 years in children screening positive versus negative for ARFID at age 4—7 years

Predictors age 0.5—3 years	Outcome age 4—7 years						
	ARFID (*n* = 49)		No ARFID (*n* = 3679)		Logistic regression		
	N	%	N	%	OR (95% CI)	*p*	Q^a^
NDP risk score^b^							
Continuous (range 0—44)	*M* = 4.54, SD = 7.37, Md = 2		*M* = 1.91, SD = 3.25, Md = 1		**1.11 (1.07, 1.16)**	**<0.0001**	—
>80th percentile (score ≥ 3)	16	33.3	620	17.0	**2.45 (1.33, 4.49)**	**0.0038**	—
>90th percentile (score ≥ 5)	10	20.8	314	8.6	**2.80 (1.38, 5.67)**	**0.0043**	—
>95th percentile (score ≥ 8)	7	14.6	180	4.9	**3.30 (1.46, 7.45)**	**0.0041**	—
>99th percentile (score ≥ 17)	4	8.3	39	1.1	**8.43 (2.89, 24.60)**	**0.0001**	—
Specific NDPs							
ESSENCE‐Q (age 2.5 years)							
Attention/concentration	13	27.7	291	8.3	**4.22 (2.20, 8.09)**	<0.0001	**0.0001**
Social interaction	8	17.4	210	6.0	**3.30 (1.52, 7.17)**	0.0025	**0.0138**
Feeding	23	50.0	1019	29.0	**2.45 (1.37, 4.39)**	0.0026	**0.0081**
General development	9	19.6	258	7.4	**3.06 (1.46, 6.41)**	0.0030	**0.0081**
Communication/language	13	28.3	464	13.2	**2.58 (1.35, 4.94)**	0.0042	**0.0081**
Sleep	11	23.9	364	10.4	**2.71 (1.36, 5.38)**	0.0044	**0.0081**
Motor development/milestones	4	8.7	130	3.7	2.47 (0.87, 6.99)	0.0886	0.1392
Behaviour (e.g., repetitive)	4	8.7	151	4.3	2.11 (0.75, 5.96)	0.1585	0.1879
Mood	7	15.2	324	9.3	1.76 (0.78, 3.97)	0.1720	0.1879
Sensory reactions	2	4.4	59	1.7	2.65 (0.63, 11.20)	0.1842	0.1879
Activity or impulsivity	8	17.4	391	11.2	1.68 (0.78, 3.62)	0.1879	0.1879
ASQ‐3 domains (ages 0.5—3 years)^c^							
Personal‐social skills	5	11.4	66	2.0	**6.45 (2.46, 16.89)**	0.0001	**0.0005**
Communication skills	6	13.3	97	2.9	**5.19 (2.15, 12.55)**	0.0003	**0.0008**
Problem‐solving skills	8	17.4	241	7.1	**2.75 (1.27, 5.96)**	0.0103	**0.0172**
Gross motor skills	6	13.0	253	7.4	1.88 (0.79, 4.47)	0.1548	0.1935
Fine motor skills	5	10.6	216	6.3	1.77 (0.69, 4.51)	0.2347	0.2347
ASQ‐3 risk score over time^d^							
0.5 years	—	—	—	—	1.01 (0.87, 1.18)	0.8503	0.8503
1 year	—	—	—	—	1.15 (0.98, 1.35)	0.0933	0.1120
1.5 years	—	—	—	—	**1.21 (1.05, 1.40)**	0.0070	**0.0105**
2 years	—	—	—	—	**1.26 (1.12, 1.42)**	0.0002	**0.0006**
2.5 years	—	—	—	—	**1.24 (1.11, 1.38)**	0.0001	**0.0006**
3 years	—	—	—	—	**1.17 (1.04, 1.32)**	0.0102	**0.0105**

Abbreviations: ASQ‐3, Ages and Stages Questionnaire; ESSENCE‐Q, Early Symptomatic Syndromes Eliciting Neurodevelopmental Clinical Examinations Questionnaire; NDPs, Neurodevelopmental problems.

^a^ *Q*‐values are the false discovery rate (FDR) adjusted *p* values using the Benjamini & Hochberg method (Benjamini & Hochberg, 1995; Benjamini & Yekutieli, 2001) correcting for the total number comparisons.

^b^ The neurodevelopmental risk score includes all of the 11 ESSENCE‐Q domains except feeding (i.e., 10 domains, each scored 0 or 1), the 5 ASQ‐3 domains (each scored 0–6), parent‐report of diagnosed developmental delay at age 1 year, and parent‐report of diagnosed motor delay, intellectual disability, and autism spectrum disorder at age 3 years (each scored 0–1), yielding a theoretical range of 0–44.

^c^ An ASQ‐3 domain was considered a neurodevelopmental problem if it was failed 2 or more out of 6 time points of measurement per domain (at 0.5, 1, 1.5, 2, 2.5 and 3 years of age).

^d^ ASQ‐3 risk score at each time point of measurement (0.5, 1, 1.5, 2, 2.5 and 3 years of age) was calculated as follows: scoring below the monitoring cut‐off on an ASQ‐3 domain–1 risk point; scoring below the referral cut‐off on an ASQ‐3 domain–2 risk points. Summed up over the five ASQ‐3 domains, this yielded an individual ASQ‐3 risk score with a theoretical range of 0–10 points per time point of measurement.

We ran exploratory logistic regressions to examine whether children with ARFID and *high* NDP risk (>90th percentile) differed from children with ARFID and *low* NDP risk (<90th percentile) in drivers of food avoidance. High NDP risk (as the dependent variable) was associated with a higher number of drivers (OR = 1.42, 95% CI 1.23–1.65) owing to a higher presence of sensory‐based food avoidance (OR = 1.70, 95% CI 1.34–2.14) and lack‐of‐interest‐based food avoidance (OR = 1.53, 95% CI 1.15–2.03), while concern‐based food avoidance was equally common (OR = 1.18, 95% CI 0.78–1.77) among those with high and low NDP risk.

**TABLE 3:** Cross‐sectional comorbidity with diagnosed NDDs in children screening positive versus negative for ARFID at age 4–7 years

	ARFID (*n* = 49)		No ARFID (*n* = 3679)		Logistic regression	
	N	%	N	%	OR (95% CI)	*P*
Autism spectrum disorder	4	8.2	62	1.7	**5.19 (1.81, 14.86)**	**0.002**
ADHD	1	2.0	49	1.3	1.54 (0.21, 11.41)	0.671
Developmental coordination disorder	1	2.0	14	0.4	5.45 (0.70, 42.31)	0.105
Intellectual disability	2	4.1	13	0.4	**12.00 (2.63, 54.66)**	**0.001**
Tic Disorders/Tourette syndrome	0	0.0	17	0.5	—	—
Specific learning disorder (e.g., dyslexia)	0	0.0	5	0.1	—	—
Any NDD	7	14.3	134	3.6	**4.41 (1.94, 10.00)**	**<0.001**

Abbreviations: ADHD, Attention‐Deficit/Hyperactivity Disorder; NDD: Neurodevelopmental disorder.

### (2) Which specific early NDPs are associated with ARFID?

Of the 11 ESSENCE‐Q domains reported at age 2.5 years, the following were significantly associated with later ARFID in simple logistic regressions after correction for 11 tests using FDR: general development, communication/language, attention/concentration, social interaction, sleep, and feeding (OR range 2.45–4.22; Table 2). Sensory reactions were not significantly associated with ARFID (OR = 2.65; 95% CI 0.63–11.20). Early feeding problems significantly predicted ARFID (OR = 2.45; 95% CI 1.37–4.39) but had the lowest odds ratio of all significant predictors. In the total sample, early feeding problems were very common (29.3%), while only 2.2% of children with early with feeding problems later screened positive for ARFID. Considered vice versa, 50% of children screening positive for ARFID had early feeding problems.

To investigate the single ASQ‐3 domains, we considered whether a child scored below the referral cut‐off *at two or more* (vs. at less than two) of the six time points of measurement between 0.5 and 3 years of age. The following domains were significantly associated with later ARFID in simple logistic regressions after multiple test correction for 5 tests: personal‐social skills, communication skills, and problem‐solving skills (OR range 2.75–6.45; Table 2).

Exploratory analysis of parent‐reported NDD diagnoses at age 4–7 years (assessed concurrently with ARFID screening) showed that children in the ARFID group had a significantly increased presence of diagnosed ASD (OR = 5.19, 95% CI 1.81–14.86) and intellectual disability (OR = 12.00, 2.63–54.66; Table 3). 14.3% of children with ARFID had *any* of the NDD diagnoses included in the survey, compared to 3.6% of children without ARFID (OR = 4.41, 95% CI 1.94–10.00). Power for these analyses was low due to the low frequency of ARFID and NDD diagnoses overall. Oppositional defiant disorder and conduct disorder were not present at all in this sample.

### (3) At what age does neurodevelopment in children with ARFID start to diverge?

**FIGURE 2:**
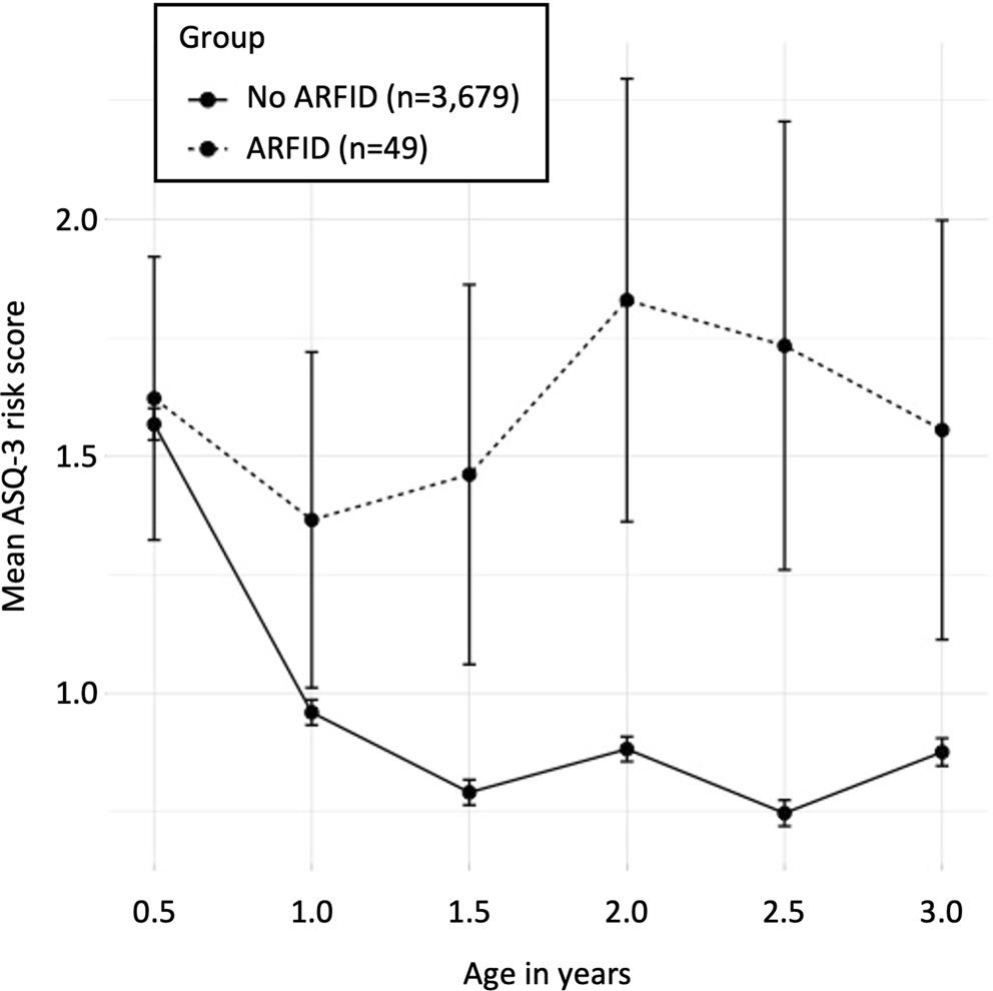
Ages and Stages Questionnaire (ASQ‐3) risk score across age by ARFID group. The *x*‐axis represents age in years. The *y*‐axis represents the mean ASQ‐3 risk score in the ARFID group (dashed line) versus the no‐ARFID group (solid line) with a theoretical range of 0–10 points per measurement point (below the monitoring cut‐off on an ASQ‐3 domain: 1 risk point; below the referral cut‐off on an ASQ‐3 domain: 2 risk points; aggregated over the five ASQ‐3 domains). Error bars represent the standard error of the mean

Lastly, we investigated group differences in the ASQ‐3 risk score trajectory over the six time points of measurement between 0.5 and 3 years of age (theoretical range ASQ‐3 risk score: 0–10 points per time point of measurement) using six simple logistic regressions. Both children with and without suspected ARFID started out at same level at 0.5 years. Descriptively, the scores started diverting after the age of 0.5 years: while the ASQ‐3 risk scores decreased and stayed at a low level in the group without suspected ARFID, scores stayed high in the ARFID group, indicating an early diversion of developmental trajectories (Figure 2). After multiple test correction, children with and without ARFID differed significantly in their ASQ‐3 risk score at ages 1.5, 2, 2.5, and 3 years (Table 2).

### DISCUSSION

The present study sought to examine the association of early NDPs with risk for ARFID in 4–7‐year‐old children. Compared to previous studies which focused on the cross‐sectional comorbidity of ARFID with NDDs, this study examined early NDPs in children with later suspected ARFID. We tested (1) to what extent early NDPs predict ARFID, (2) which *specific* early NDPs are associated with ARFID, and (3) at what age neurodevelopment in children with ARFID starts to diverge. Overall, results confirmed the previously observed association between ARFID and NDPs/NDDs. Specific results will be discussed in turn.

In line with our hypothesis, we found that children with early NDPs (not including early feeding problems) were at *increased risk* for ARFID. Specifically, risk of later ARFID was circa three times higher in children with several NDPs (i.e., at high risk of NDDs). For example, circa 3.1% of children scoring in the highest risk decile of the NDP risk score developed ARFID, as opposed to 1.1% of children scoring below the highest risk decile of the NDP risk score. Early NDPs significantly predicted later ARFID, but the total explained variance was small (3.2%). As no previous studies have examined the longitudinal association between early NDPs and later ARFID, it is not possible to compare these results. Studies in clinical samples might produce stronger associations between ARFID and NDDs, as individuals with higher comorbidity might have a higher clinical severity leading to treatment‐seeking and their inclusion into studies on clinical samples. For instance, an exploratory analysis in this sample showed that children with suspected ARFID and *high* NDP risk on average had a higher number of drivers of food avoidance than children with suspected ARFID and *low* NDP risk, potentially indicating that NDDs are associated with a higher severity of ARFID. In addition, as a previous attrition analysis showed, our sample might have been slightly healthier than the average Japanese population, further weakening the detectable association between ARFID and NDPs. Alternatively, we may have underestimated the presence of NDPs, since they were assessed at a very early age (0.5–3 years) and NDPs sometimes do not become obvious before later childhood or adolescence (Hosozawa et al., 2020; Mandy et al., 2018).

Second, we tested the association of *specific* early NDPs with ARFID. In line with our hypothesis, specifically early NDPs related to ASD (communication/language, social interaction), ADHD (attention/concentration), and intellectual disability (general development) were associated with ARFID, as well as problems related to sleep. This is in line with previous research reporting an overrepresentation of ASD, ADHD and developmental delay in ARFID (Lieberman et al., 2019; Nicely et al., 2014; Norris et al., 2021; Reilly et al., 2019), as well as with the overrepresentation of ASD and intellectual disability diagnoses at follow‐up in the present sample (note that the present sample was largely too young for ADHD diagnoses, as evidenced by the low prevalence of 1.3%). Surprisingly, early problems with sensory reactions were not significantly associated with later ARFID. Considering that 51% of the children with suspected ARFID showed sensory‐based food avoidance, we would have expected to see increased problems with sensory reactions early on. The observed non‐significant association could potentially be explained by the following. The ESSENCE‐Q item for concerns around sensory reactions is very unspecific; it asks about hyper‐ as well as hyposensitivity to all kinds of sensory impressions [“Have you (or anybody else, who?) been concerned for more than a few months regarding the child's sensory reactions (e.g., touch, sound, light, smell, taste, heat, cold, pain)?”]. Furthermore, sensory sensitivity to food characteristics might lead to extremely cautious eating behaviour (i.e., food avoidance) only in combination with low tolerance of variation/surprise and high level of risk avoidance.

We further hypothesized that most children with suspected ARFID would show feeding problems early on. We found that 50% of children screening positive for ARFID had early feeding problems, indicating that in half of the children, the onset of ARFID might have been after the age of 2.5 years. In line with previous research on eating behaviour in early childhood (Taylor et al., 2015), feeding problems at age 2.5 years were very common (29.3% in the whole sample). However, the proportion of children with early feeding problems who were later suspected of having ARFID was small (2.2%), which shows that early feeding problems as reported by parents in the ESSENCE‐Q are unspecific and not a good predictor of later ARFID [“Have you (or anybody else, who?) been concerned for more than a few months regarding the child's feeding?”]. Our results also showed that this prediction could be significantly improved by including the whole range of NDPs assessed in this study, which implies that early NDPs can aid in identifying risk of ARFID in children with and *without* early feeding problems.

Lastly, exploratory analysis of ASQ‐3 risk score trajectories from age 0.5–3 years showed an early diversion of developmental trajectories (after the age of 1 year) in children with suspected ARFID. This is an interesting finding indicating that closely monitoring feeding problems in children with NDPs might be an important opportunity to prevent the development of ARFID.

### Strengths and limitations

Our study has several strengths. To our knowledge, this is the first study to examine prospectively collected data on a broad range of early NDPs in children screening positive for ARFID in a large non‐clinical sample. This enabled us to study the occurrence and predictive power of early NDPs in a group with suspected ARFID that is likely to be representative of 4‐7‐year‐old Japanese children with ARFID, as it also includes those who do not seek treatment and hence are not part of clinical samples. By studying very young children, we provide data on an age group that has been largely neglected in ARFID research so far. Furthermore, we explicitly excluded early feeding problems from the NDP risk score to identify their separate contributions.

Several limitations must be considered. First, in the present study we screened for ARFID using a newly developed parent‐reported screening tool which still has to be more fully validated against clinical ARFID diagnoses. In a previous study using this screening tool we found promising initial evidence of convergent validity with a range of measures assessing restrictive type eating as well as with weight and height (Dinkler et al., 2022). Second, although the total sample was relatively large, the number of children with ARFID was low, resulting in low power for the multiple regression analyses. Future research studying ARFID in non‐clinical samples needs to employ even larger samples. Third, information on NDPs and diagnosed NDDs was collected through parent‐reports only. Parents might not always be aware of NDPs, or they might underreport diagnoses due to associated stigma. On the other hand, parents might be over‐worried and therefore overreport symptoms, whereas overreporting of diagnoses seems unlikely. Optimally, NDPs and NDDs should be clinically ascertained, which is, however, not always feasible, especially in epidemiological research including large samples like this one. Lastly, the response rate was 56.6%. Although the initially enrolled JECS cohort is representative for the Japanese population (Michikawa et al., 2018), our and others' attrition analyses showed that responders are slightly healthier and more affluent than non‐responders (Dinkler et al., 2022; Kigawa et al., 2019). It is therefore possible that children with ARFID in general, and specifically those with a higher disease burden or comorbid NDDs, were less likely to be included in our sample, which might have resulted in an underestimation of the association between ARFID and early NDPs, while an overestimation is unlikely.

## CONCLUSION

The present study showed that circa 3% of the children at high risk for NDDs in preschool age screened positive for ARFID later on (between age 4 and 7 years), which made them three times more likely to have suspected ARFID than children at low NDP risk. Specific NDPs that were present at a higher rate in children with suspected ARFID reflected the increased prevalence of ASD, ADHD, developmental delay, and intellectual disability observed in clinical samples with ARFID. Our results largely mirror the previously reported overrepresentation of NDPs/NDDs in individuals with ARFID. Considering the early onset of ARFID in many individuals and the observed comorbidity with NDDs, future research should investigate whether ARFID itself can be considered part of the NDP/NDD spectrum, at least in those with early onset and high NDD comorbidity. Our results also imply that, while early feeding problems are common and rarely develop into ARFID, they should be monitored closely in children with high NDP risk, which can easily be screened for. In doing so, we might be able to prevent the worsening of eating pathology until full criteria for ARFID are met.

## AUTHOR CONTRIBUTIONS

**Lisa Dinkler:** Conceptualization; Data curation; Formal analysis; Funding acquisition; Investigation; Methodology; Project administration; Visualization; Writing – original draft; Writing – review & editing. **Kahoko Yasumitsu‐Lovell:** Data curation; Investigation; Project administration; Writing – review & editing. **Masamitsu Eitoku:** Writing – review & editing. **Mikiya Fujieda:** Funding acquisition; Writing – review & editing. **Narufumi Suganuma:** Project administration; Resources; Writing – review & editing. **Yuhei Hatakenaka:** Resources; Writing – review & editing. **Nouchine Hadjikhani:** Writing – review & editing. **Rachel Bryant‐Waugh:** Supervision; Writing – review & editing. **Maria Råstam:** Conceptualization; Funding acquisition; Supervision; Writing – review & editing. **Christopher Gillberg:** Conceptualization; Funding acquisition; Methodology; Resources; Supervision; Writing – review & editing.

## CONFLICT OF INTEREST

The authors have declared that they have no competing or potential conflicts of interest.

## ETHICS STATEMENT

The study was approved by the Ethics Committee at Kochi Medical School (ERB‐102925 and ERB‐104083). Informed consent was obtained from all participants.

## Supporting information

Table S1

## Data Availability

Data are unsuitable for public deposition due to ethical restrictions and legal framework of Japan.
