# Supplementary material for: Early neurodevelopmental problems and risk for avoidant/restrictive food intake disorder (ARFID) in 4‐7‐year‐old children: A Japanese birth cohort study
Source: JCPP Adv. 2022 Aug 7;2(3):e12094. doi: 10.1002/jcv2.12094 (PMC10242837; doi:10.1002/jcv2.12094)
Supplement: Supplementary file 1 — Table S1 [file JCV2-2-e12094-s001.docx]

**Table S1** *Items used to screen the DSM-5 diagnostic criteria for avoidant/restrictive food intake disorder (ARFID), including the prevalence of criteria A1-A4 and drivers of food avoidance in the ARFID group*

| Criterion | Item | Response options^a^ | Prevalence in ARFID group in % |
| --- | --- | --- | --- |
| A - Avoidance or restriction of food intake | Do you think your child has an eating or feeding disturbance characterized by avoidance or restriction of food intake? (avoidance and restriction can relate to the range of foods eaten as well as the overall amount eaten) | **Yes, currently** Yes, earlier  No, never | 100.0 |
| A1 - Significant weight loss (or failure to grow/gain weight) | Over the past 3 months has there been concern that your child has not gained weight or grown as he/she should? | **Yes, currently** Yes, earlier  No, never | 49.0 |
| A2 - Significant nutritional deficiency | Has your child been identified by a health professional as having any nutritional deficiency? | **Yes, currently** Yes, earlier  No, never | 4.1 |
| A3 - Dependence on enteral feeding or oral nutritional supplements | a) Has your child been prescribed dietary supplements (e.g., vitamins, minerals) to address nutritional deficiencies? | **Yes, currently** Yes, earlier  No, never | 6.1 |
|  | b) Did your child ever need nutritional supplement drinks (or other high-energy drinks) to be able to maintain/gain weight? | **Yes, currently** Yes, earlier  No, never |  |
| A4 - Marked interference with psychosocial functioning | a) Do you believe that your child's *current* eating pattern causes any distress for your child? | **Yes, a lot**  Yes, somewhat  Not at all | 63.3 |
|  | b) Does your child's *current* eating pattern interfere with his/her social functioning (e.g., attending preschool, affecting meals in preschool, making friends, play, activities)? | **Yes, a lot**  Yes, somewhat  Not at all |  |
| C - Eating disturbance not attributable to weight/shape concerns | My child says that he/she feels fat, even if other people do not agree with him/her. (*item from Eating Disorder in Youth-Questionnaire, EDY-Q*) | **Never**  **Rarely**  **Sometimes**  Often  Always | ---- |
| D – Eating disturbance not attributable to concurrent medical condition | If your child has any problems with weight, growth or nutrition, is this primarily due to a current medical problem?  Medical problem: (specify) | Yes  **No** | ---- |
| Driver - Lack of interest in food or eating | My child enjoys eating. *(item from* *Behavioral Pediatric Feeding Assessment Scale, BPFAS; reverse item)* | **Never**  **Rarely**  **Sometimes**  Often  Always | 63.3 |
| Driver – Sensory-based avoidance | My child dislikes to eat food with a specific smell, taste, appearance, temperature, or a certain consistency/texture (e.g., crispy or soft). | Never  Rarely  **Sometimes**  **Often**  **Always** | 51.0 |
| Driver – Concern about aversive consequences of eating | My child is afraid of eating because of worries about what might happen (e.g., choking, vomiting, stomach aches, diarrhoea, or allergic reactions etc.). | Never  Rarely  **Sometimes**  **Often**  **Always** | 14.3 |

^a^ Response option(s) required to meet the respective criterion printed in bold.

**Screening algorithm:** A + (A1 or A2 or A3-a or A3-b or A4-a or A4-b) + C + D
